# Supplementary material for: Ternary Liquid-Crystalline Mixture Showing Broad Antiferroelectric Smectic CA* and Glassy Hexatic Smectic XA* Phases
Source: J Phys Chem B. 2026 Sep 8;130(37):9655–67. doi: 10.1021/acs.jpcb.6c02873 (PMC13592077; doi:10.1021/acs.jpcb.6c02873)
Supplement: Supplementary file 1 [file jp6c02873_si_001.pdf]

Ternary Liquid Crystalline Mixture Showing Broad Antiferroelectric Smectic C_A* and Glassy Hexatic Smectic X_A* Phases

Aleksandra Deptuch [a,*], Anna Drzewicz [a], Marcin Piwowarczyk [a], Michał Czerwiński [b], Mateusz Filipow [b], Mateusz Pączek [c], Ewa Juszyńska-Gałązka [a,d]

[a] Institute of Nuclear Physics, Polish Academy of Sciences, Radzikowskiego 152, PL-31342 Kraków, Poland

[b] Institute of Chemistry, Military University of Technology, Kaliskiego 2, PL-00908 Warsaw, Poland

[c] Faculty of Chemistry, Jagiellonian University, Gronostajowa 2, PL-30387 Kraków, Poland

[d] Research Center for Thermal and Entropic Science, Graduate School of Science, Osaka University, 560-0043 Osaka, Japan

*corresponding author, aleksandra.deptuch@ifj.edu.pl

Supporting Information

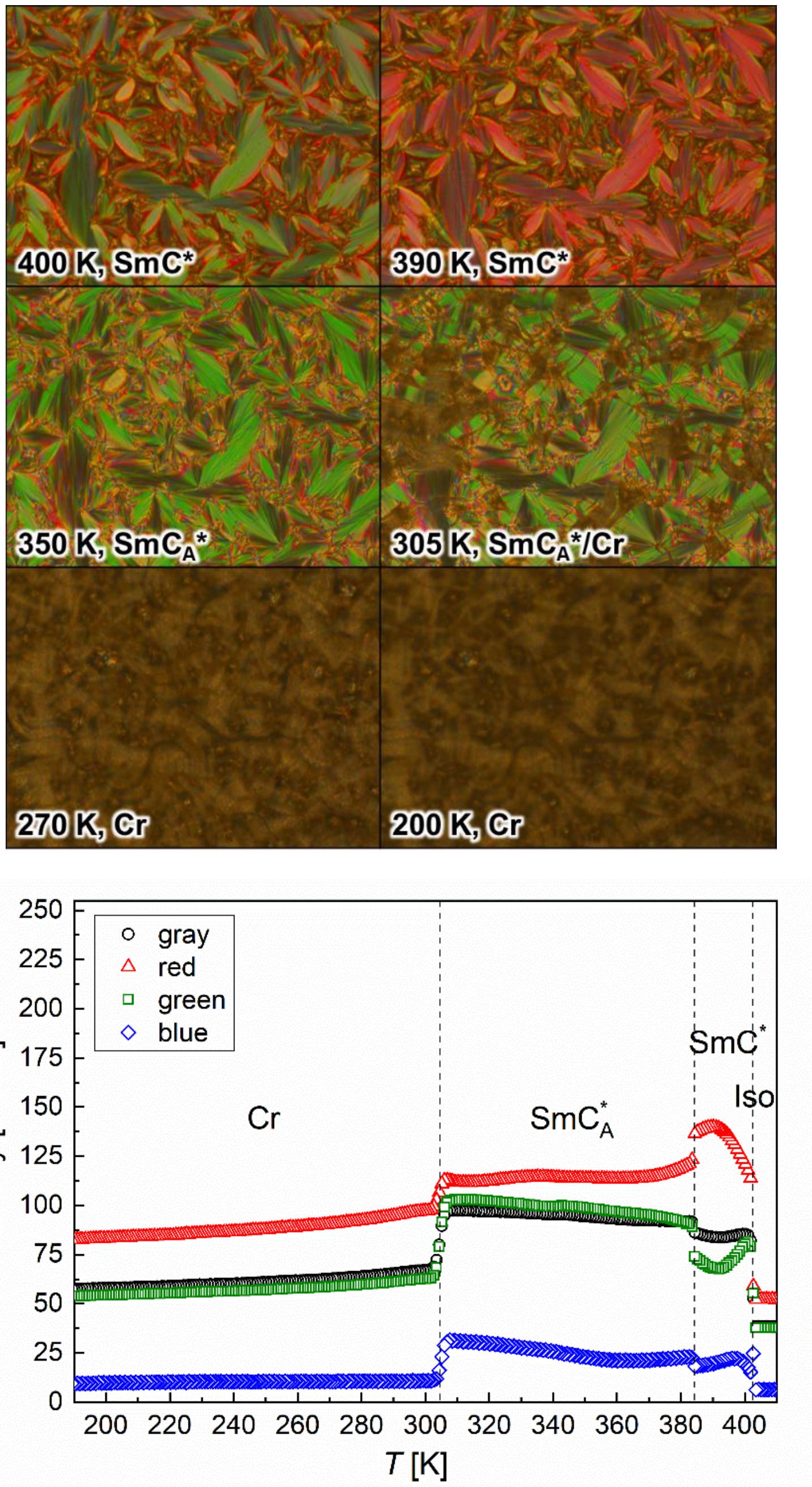

Figure S1. POM textures (622 × 466 μm^2) of 3F2HPhH6 collected at the 10 K/min cooling rate in the transmission mode. The plot below shows the red, green, blue components and weighted total intensity of each texture.

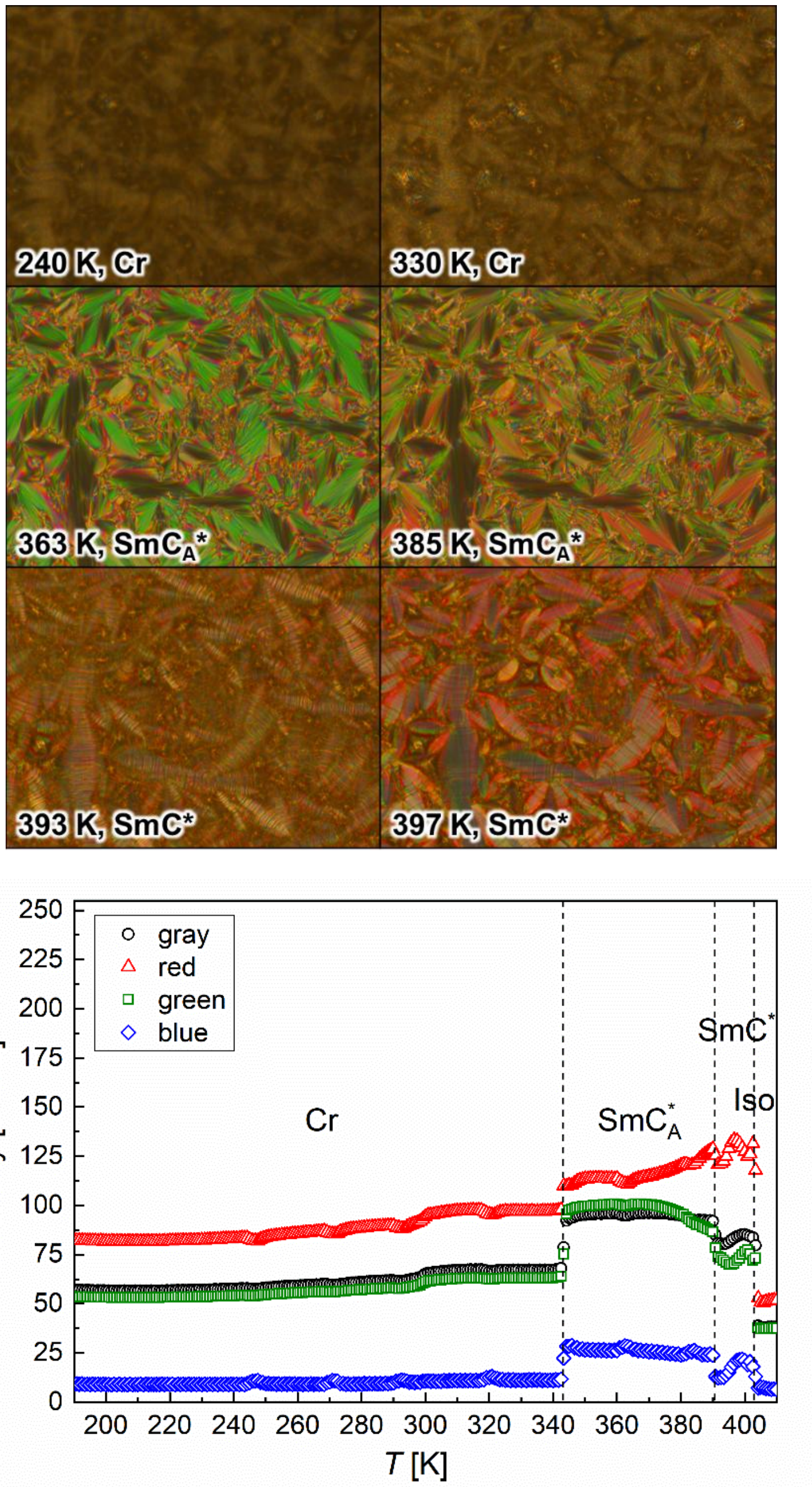

Figure S2. POM textures (622 × 466 μm^2) of 3F2HPhH6 collected at the 10 K/min heating rate in the transmission mode. The plot below shows the red, green, blue components and weighted total intensity of each texture.

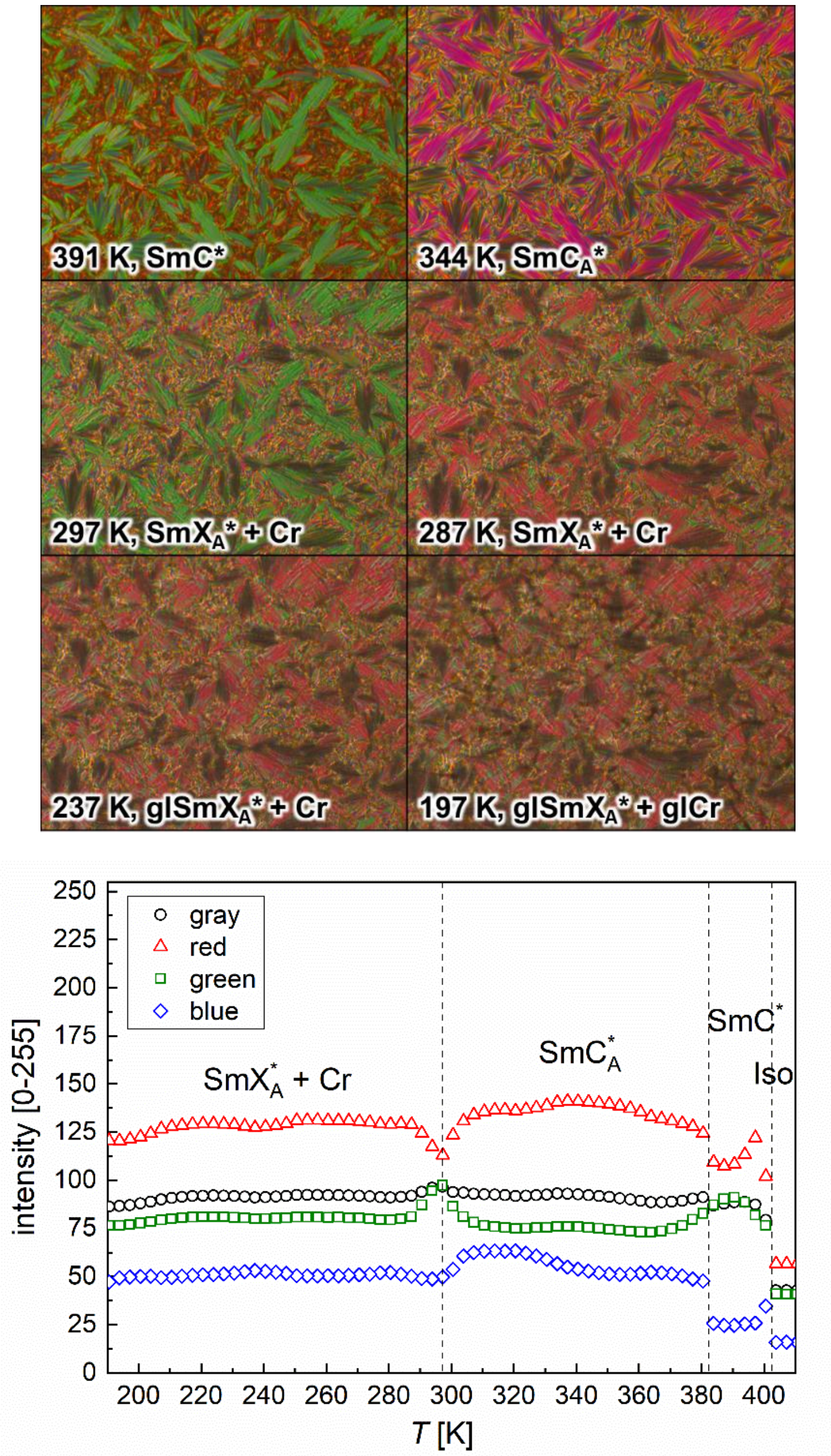

Figure S3. POM textures (622 × 466 μm^2) of 3F2HPhH6 collected at the 40 K/min cooling rate in the transmission mode. The plot below shows the red, green, blue components and weighted total intensity of each texture.

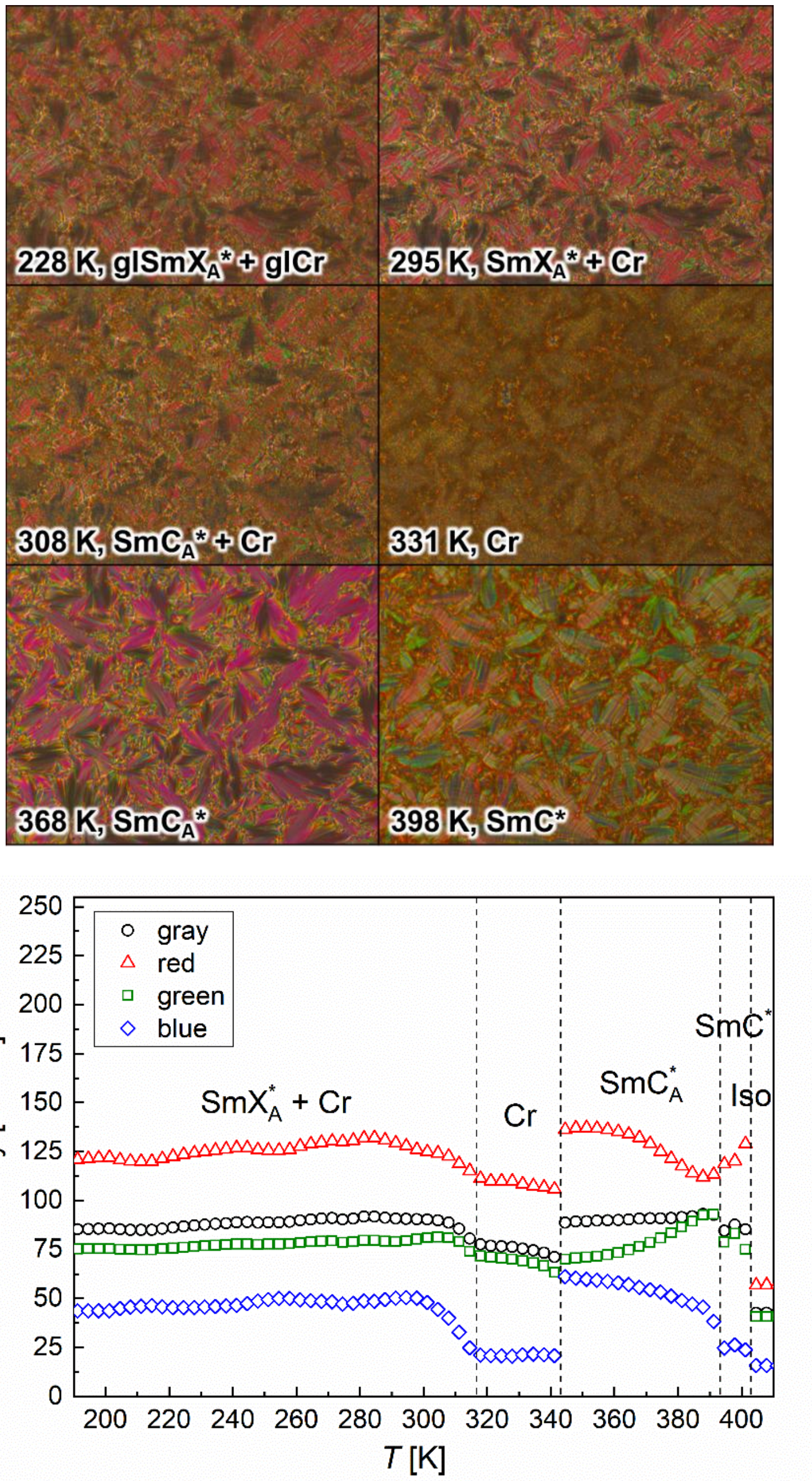

Figure S4. POM textures (622 × 466 μm²) of 3F2HPhH6 collected at the 40 K/min heating rate in the transmission mode. The plot below shows the red, green, blue components and weighted total intensity of each texture.

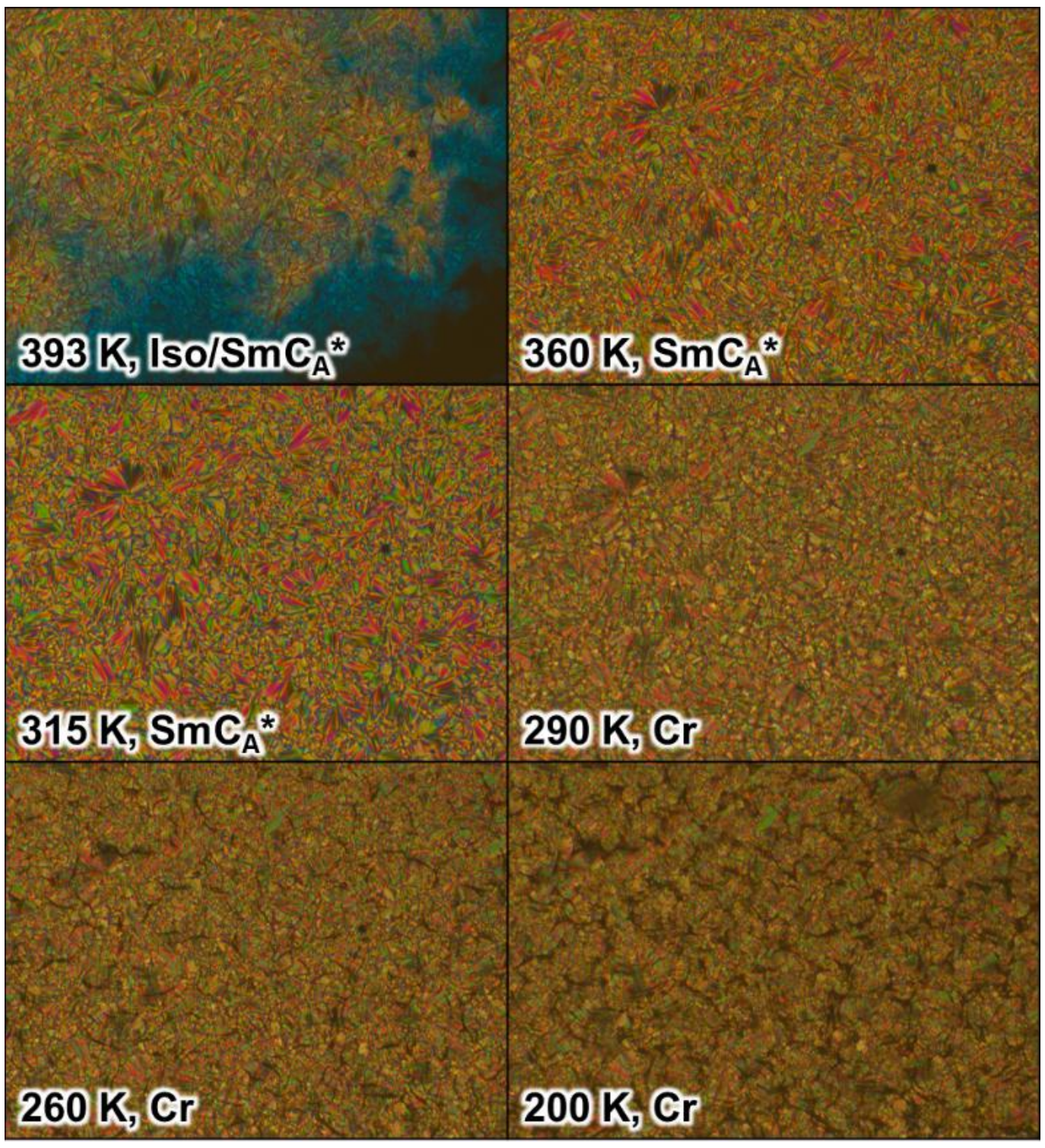

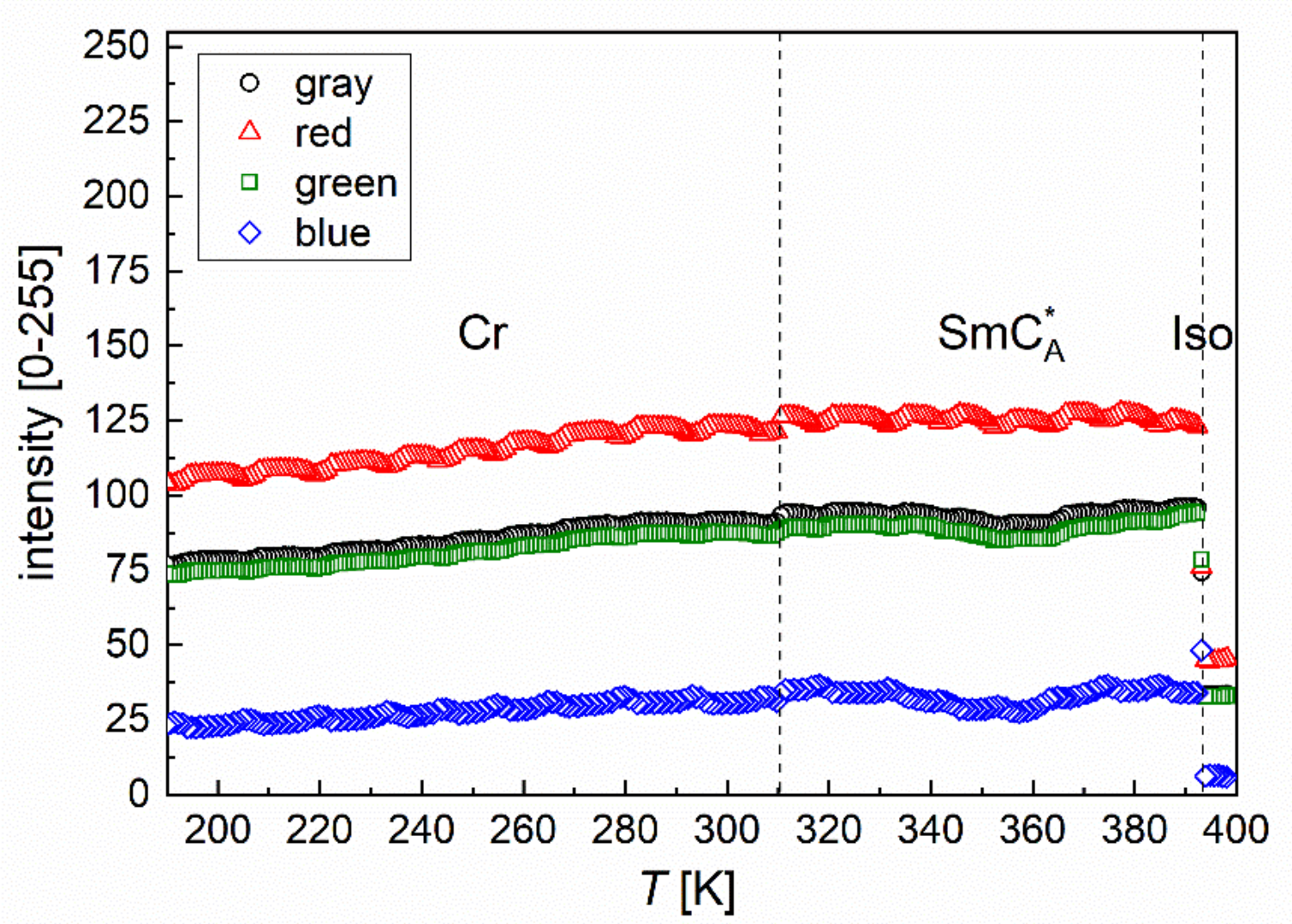

Figure S5. POM textures (622 × 466 μm^2) of 3F3HPhH6 collected at the 10 K/min cooling rate in the transmission mode. The plot below shows the red, green, blue components and weighted total intensity of each texture.

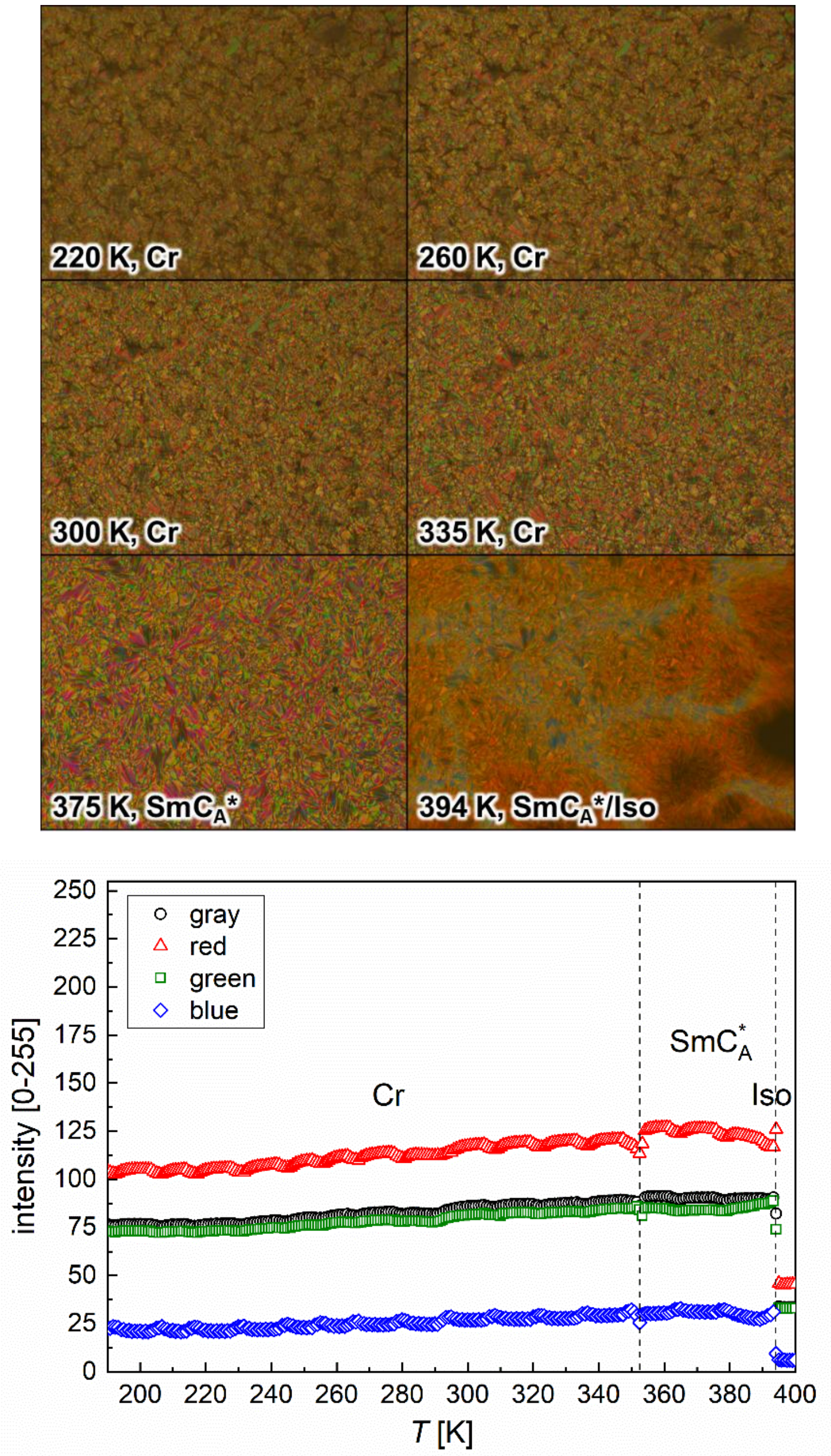

Figure S6. POM textures (622 × 466 μm^2) of 3F3HPhH6 collected at the 10 K/min heating rate in the transmission mode. The plot below shows the red, green, blue components and weighted total intensity of each texture.

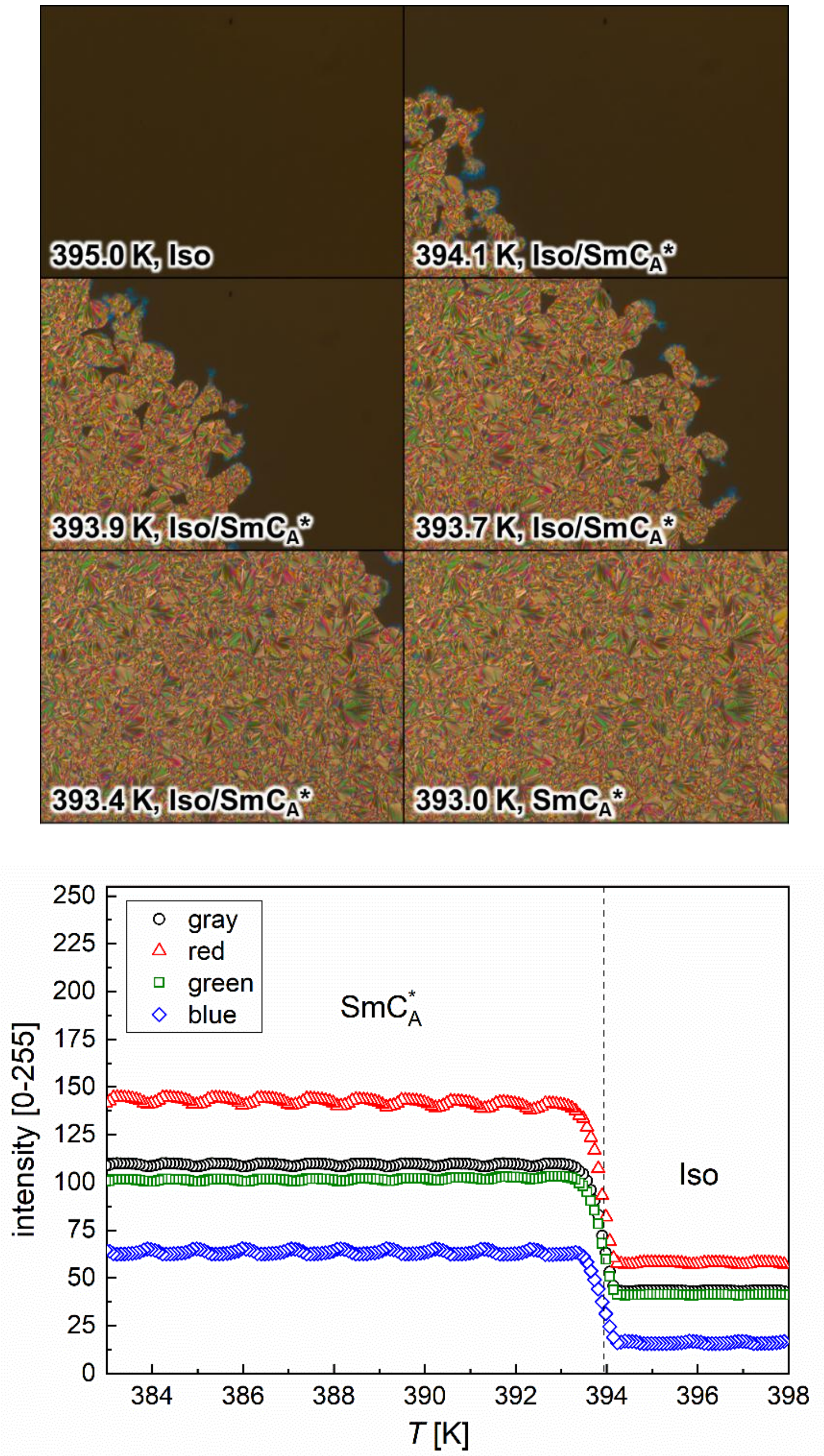

Figure S7. POM textures (622 × 466 μm^2) of 3F3HPhH6 collected at the 1 K/min cooling rate in the transmission mode. The plot below shows the red, green, blue components and weighted total intensity of each texture.

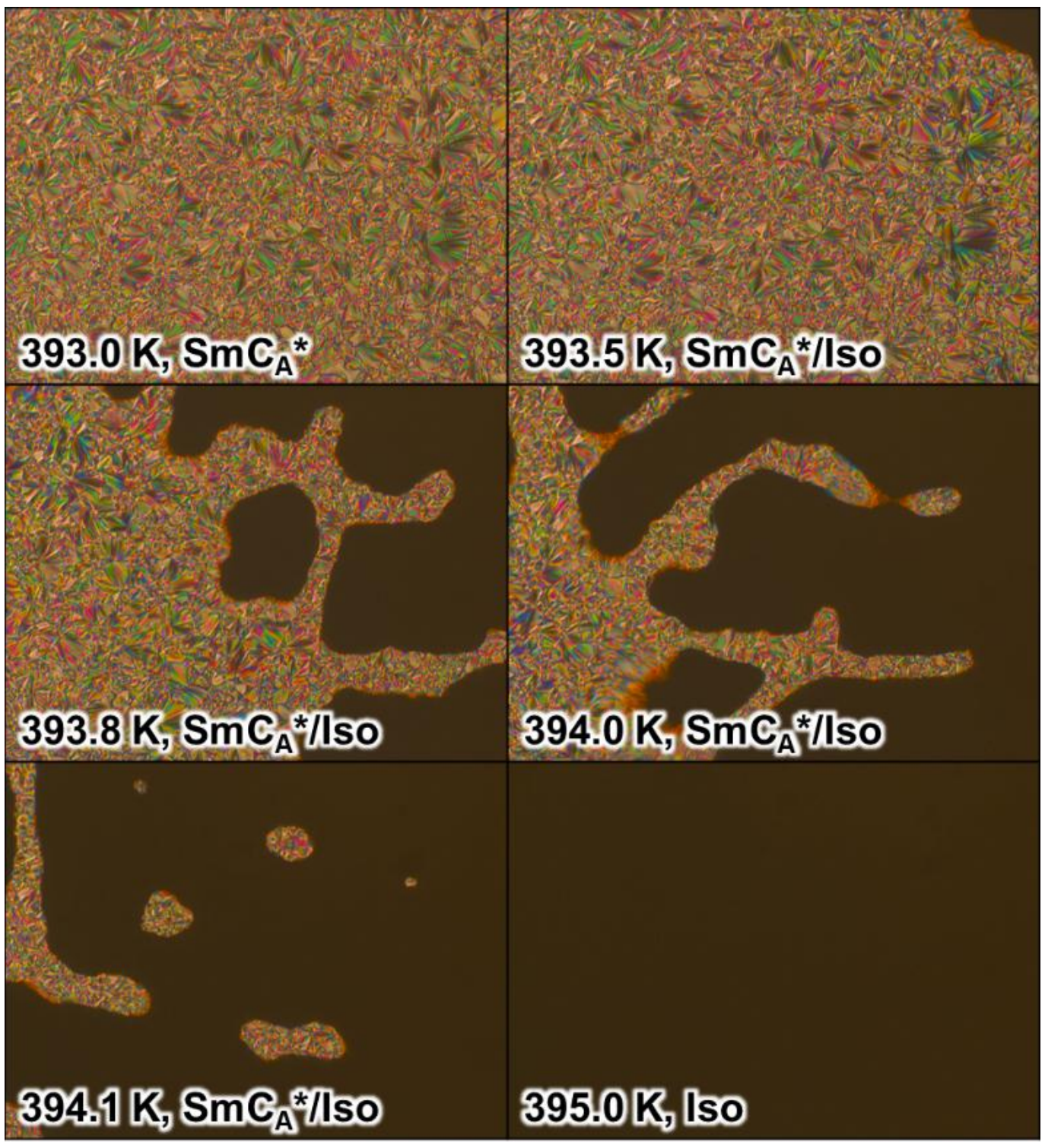

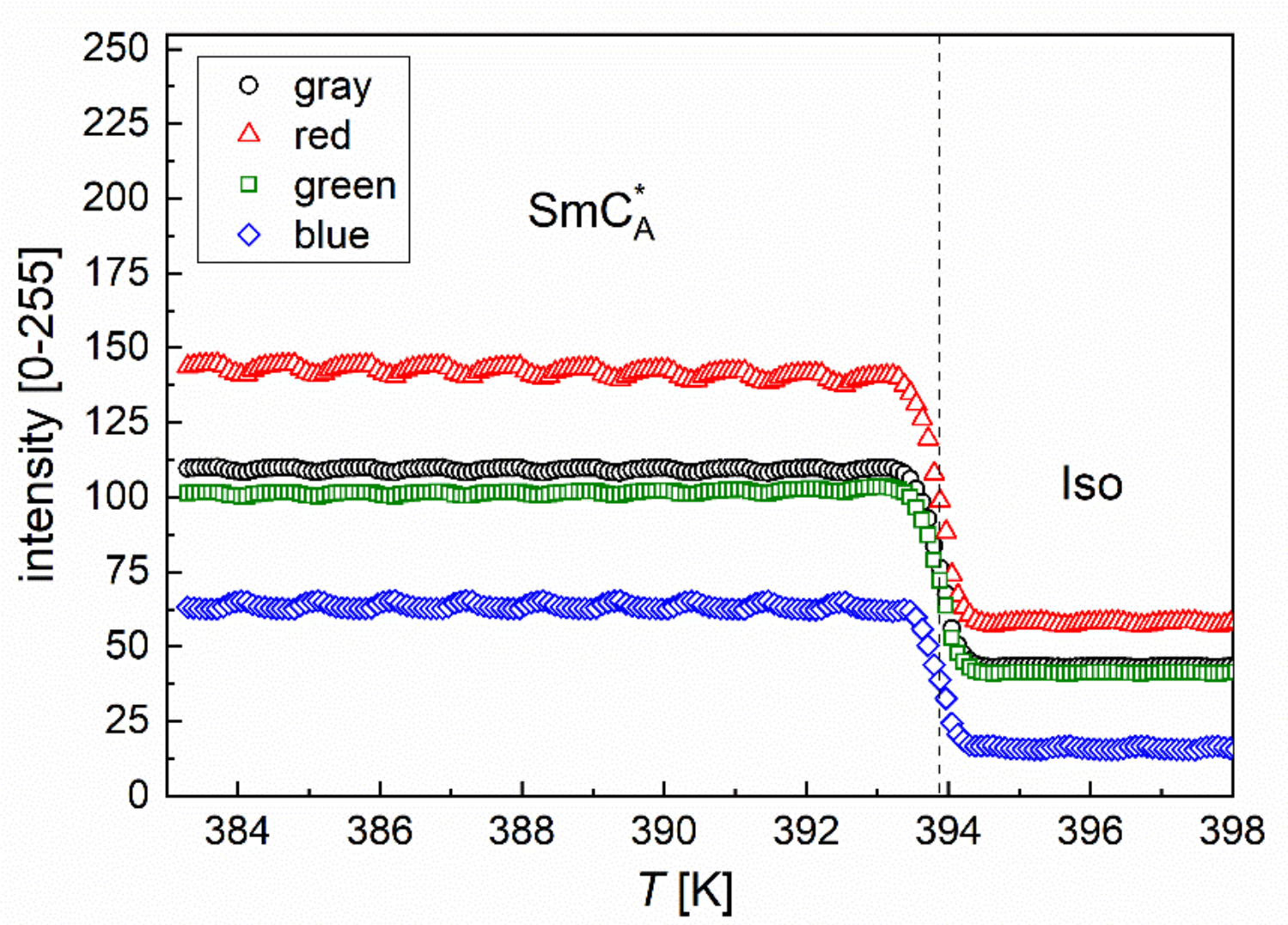

Figure S8. POM textures (622 × 466 μm^2) of 3F3HPhH6 collected at the 1 K/min heating rate in the transmission mode. The plot below shows the red, green, blue components and weighted total intensity of each texture.

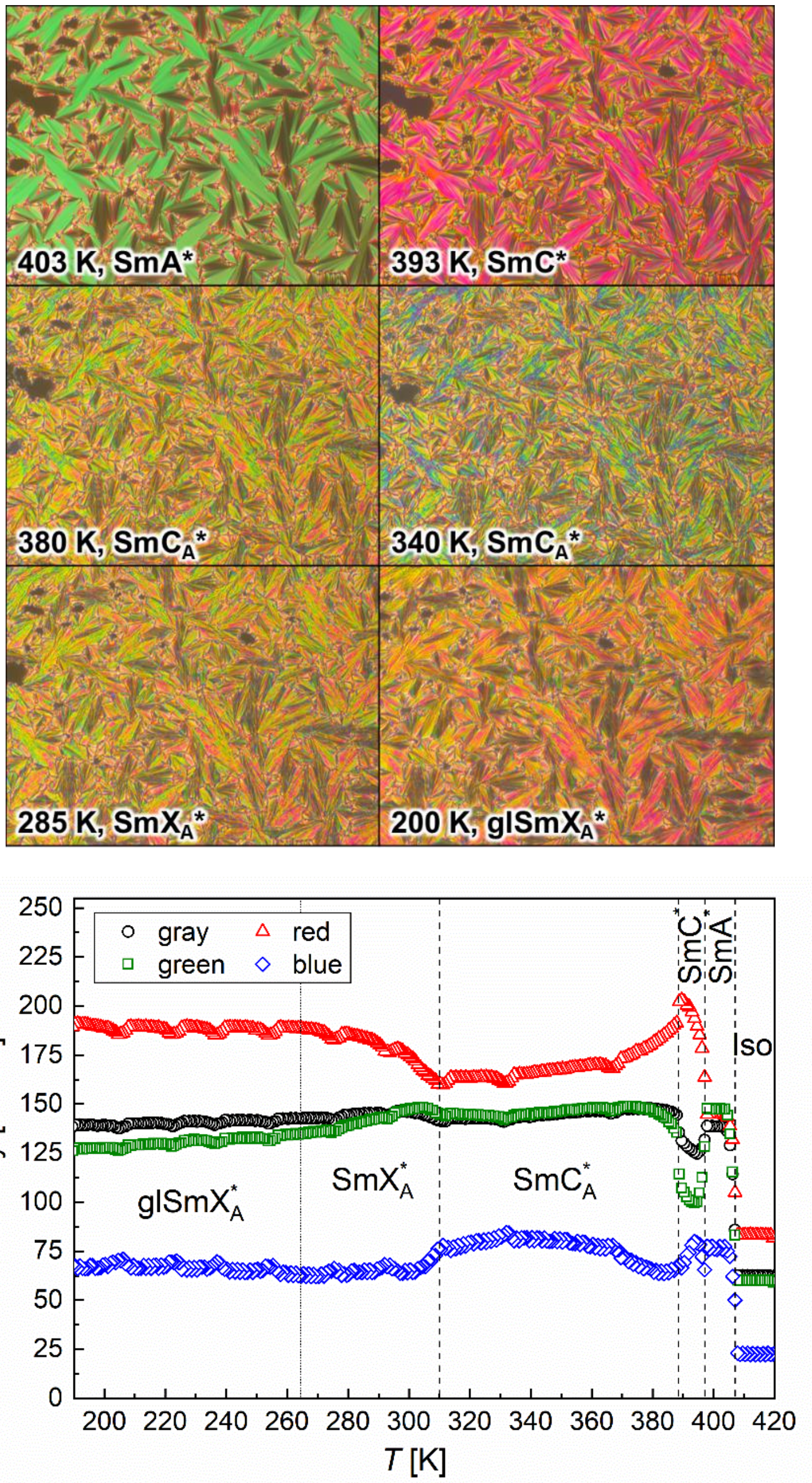

Figure S9. POM textures (622 × 466 μm^2) of MIX23HH6 collected at the 10 K/min cooling rate in the transmission mode. The plot below shows the red, green, blue components and weighted total intensity of each texture. The glass transition temperature is based on the corresponding DSC results.

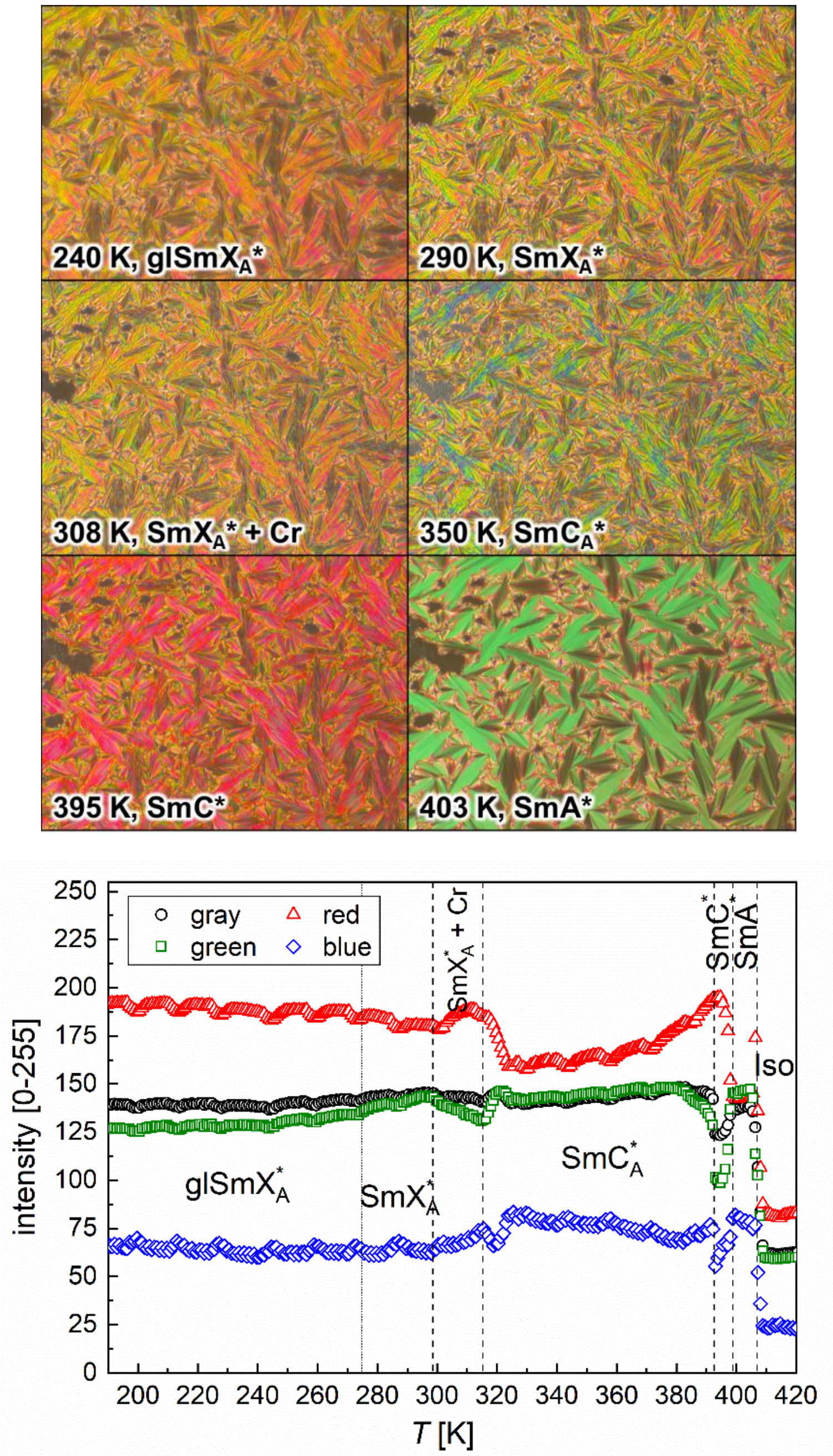

Figure S10. POM textures (622 × 466 μm^2) of MIX23HH6 collected at the 10 K/min heating rate in the transmission mode. The plot below shows the red, green, blue components and weighted total intensity of each texture. The glass transition temperature is based on the corresponding DSC results.

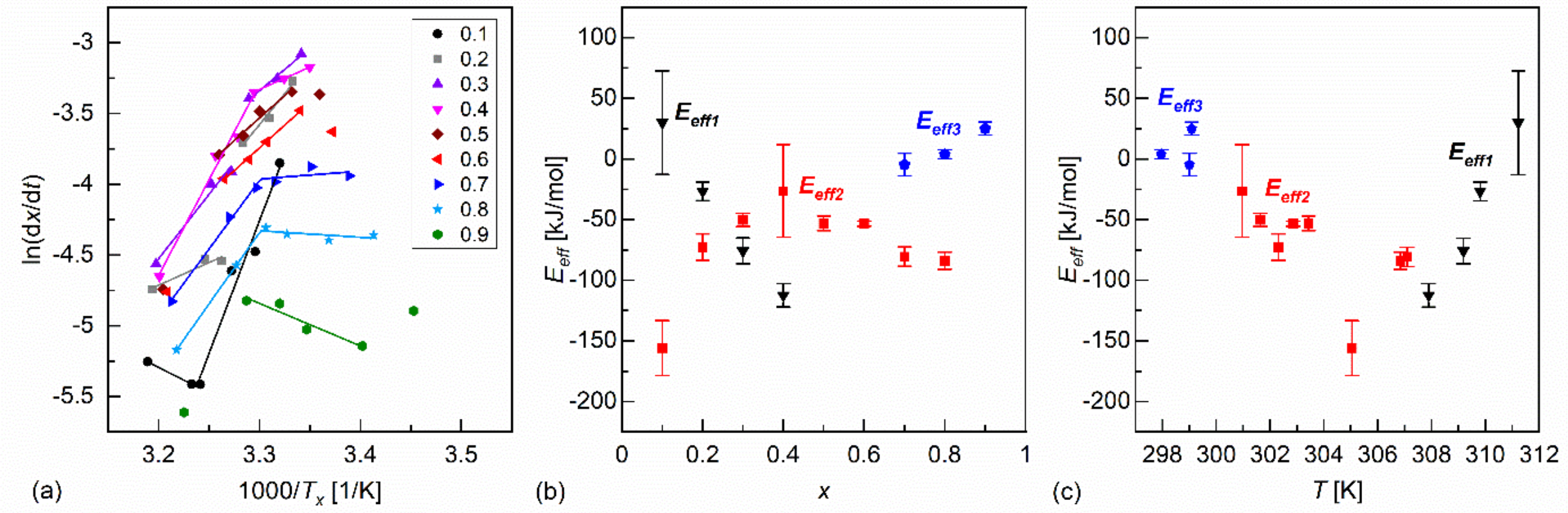

Figure S11. The melt crystallization of 3F2HPhH6 analyzed by the isoconversional method: activation plot (a), effective activation energy vs. conversion degree (b), effective activation energy vs. average temperature.

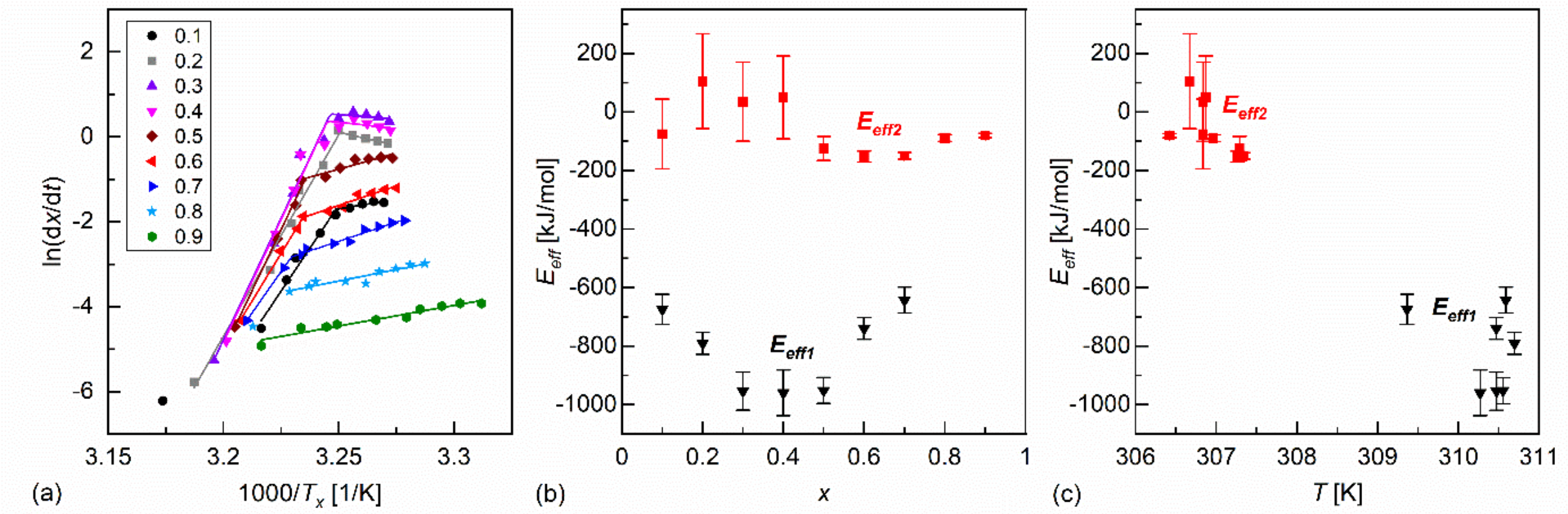

Figure S12. The melt crystallization of 3F3HPhH6 analyzed by the isoconversional method: activation plot (a), effective activation energy vs. conversion degree (b), effective activation energy vs. average temperature.

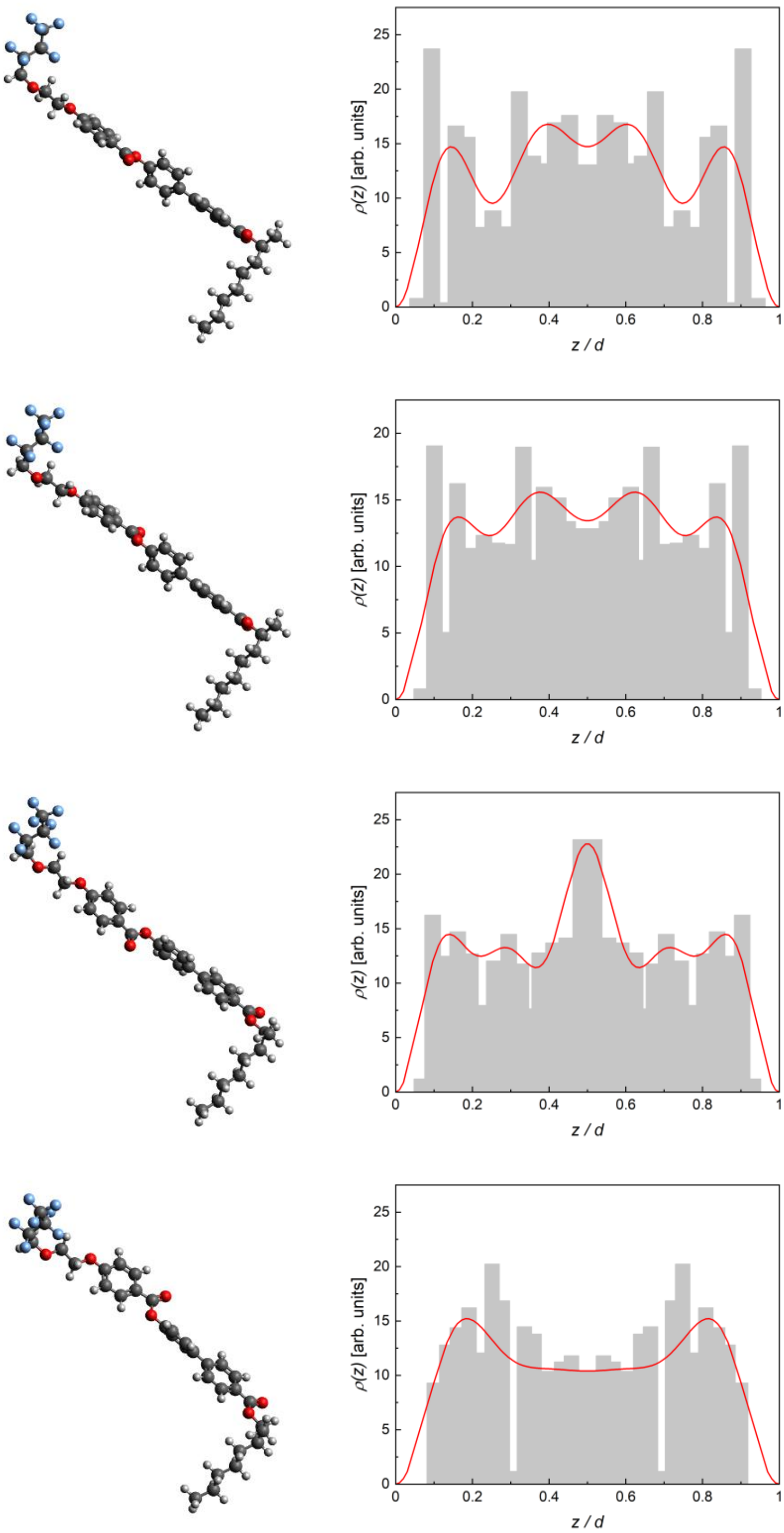

Figure S13. Models of 3F2HPhH6 optimized at the B3LYP-D3(BJ)/6-31+Gd level and corresponding electron density profiles along the smectic layer normal. Lines are fitting results of Equation (2) from the main text.

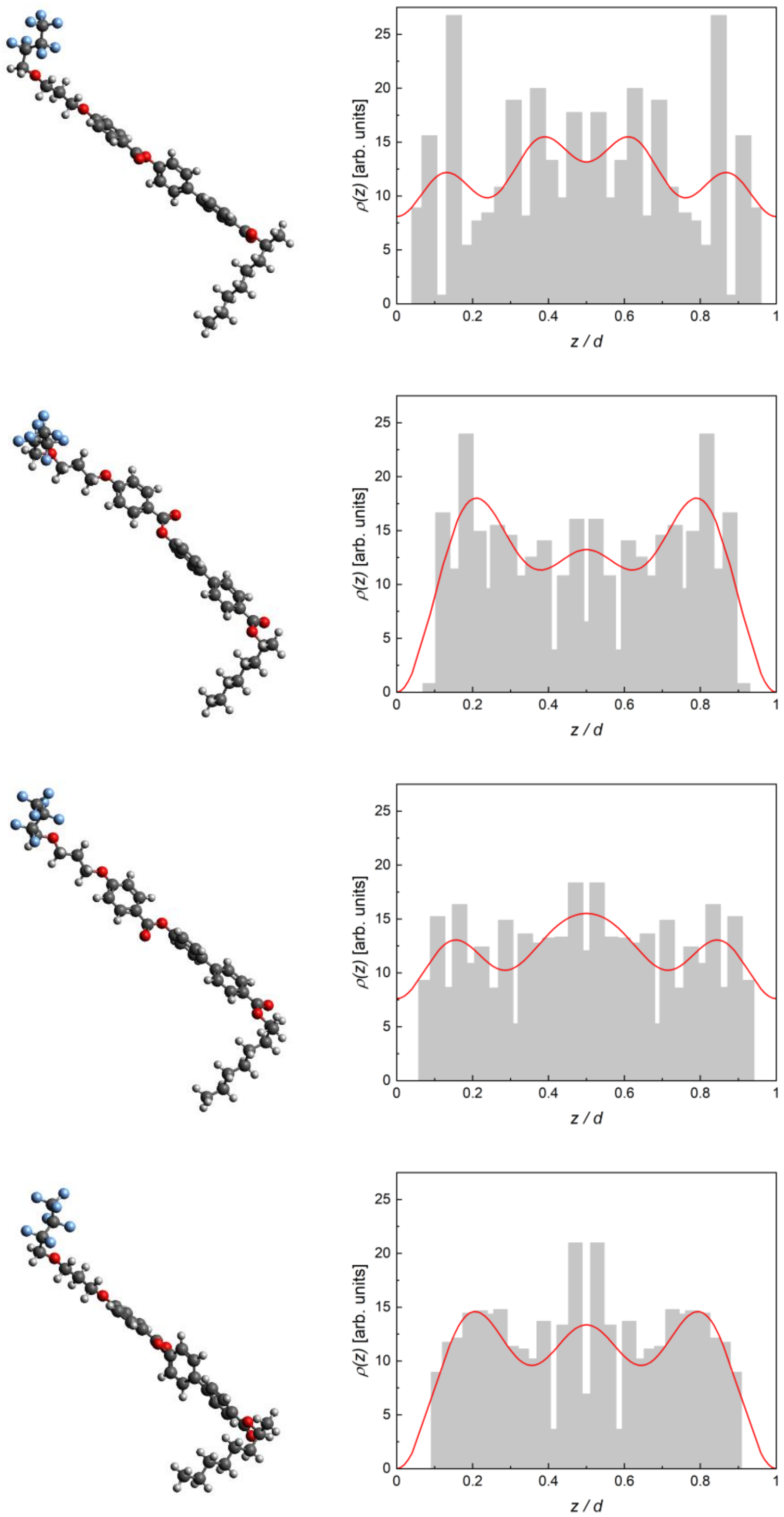

Figure S14. Models of 3F3HPhH6 optimized at the B3LYP-D3(BJ)/6-31+Gd level and corresponding electron density profiles along the smectic layer normal. Lines are fitting results of Equation (2) from the main text.

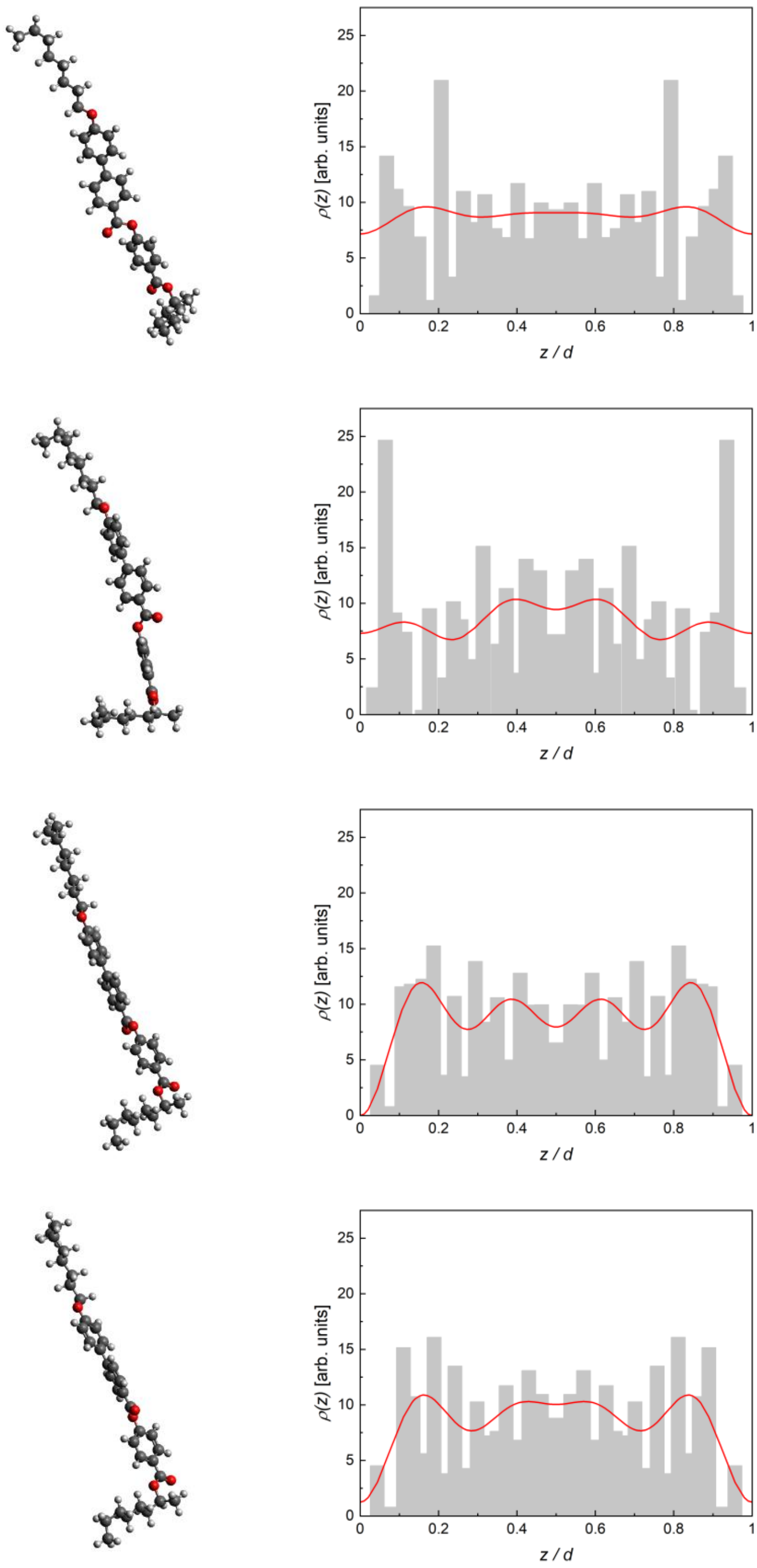

Figure S15. Models of MHPOBC optimized at the B3LYP-D3(BJ)/6-31+Gd level and corresponding electron density profiles along the smectic layer normal. Lines are fitting results of Equation (2) from the main text.

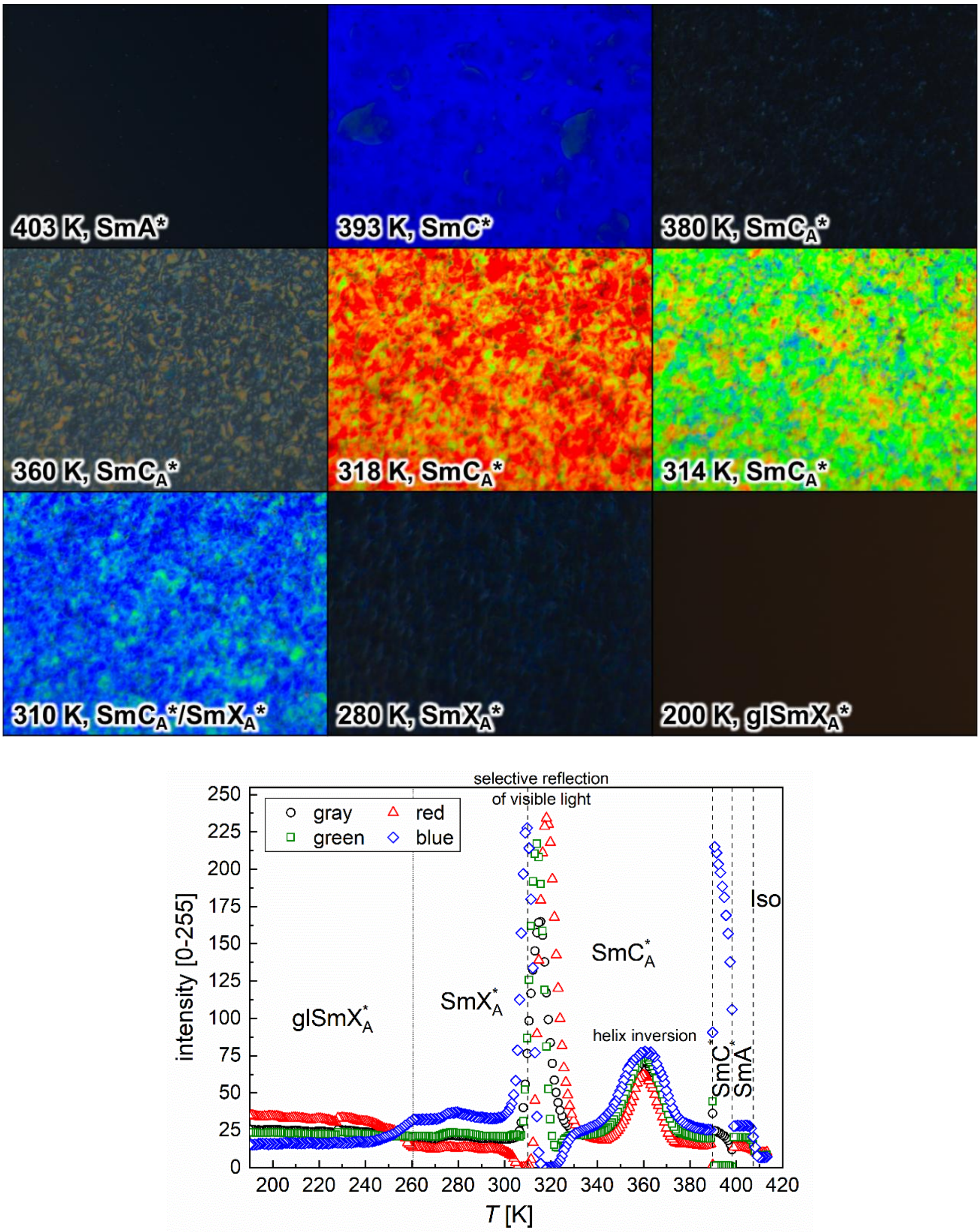

Figure S16. POM textures (622 × 466 μm^2) of MIX23HH6 collected at the 10 K/min cooling rate in the reflection mode. The plot below shows the red, green, blue components and weighted total intensity of each texture. The SmC_A* → SmX_A* transition temperature is based on the data from Figure S9.

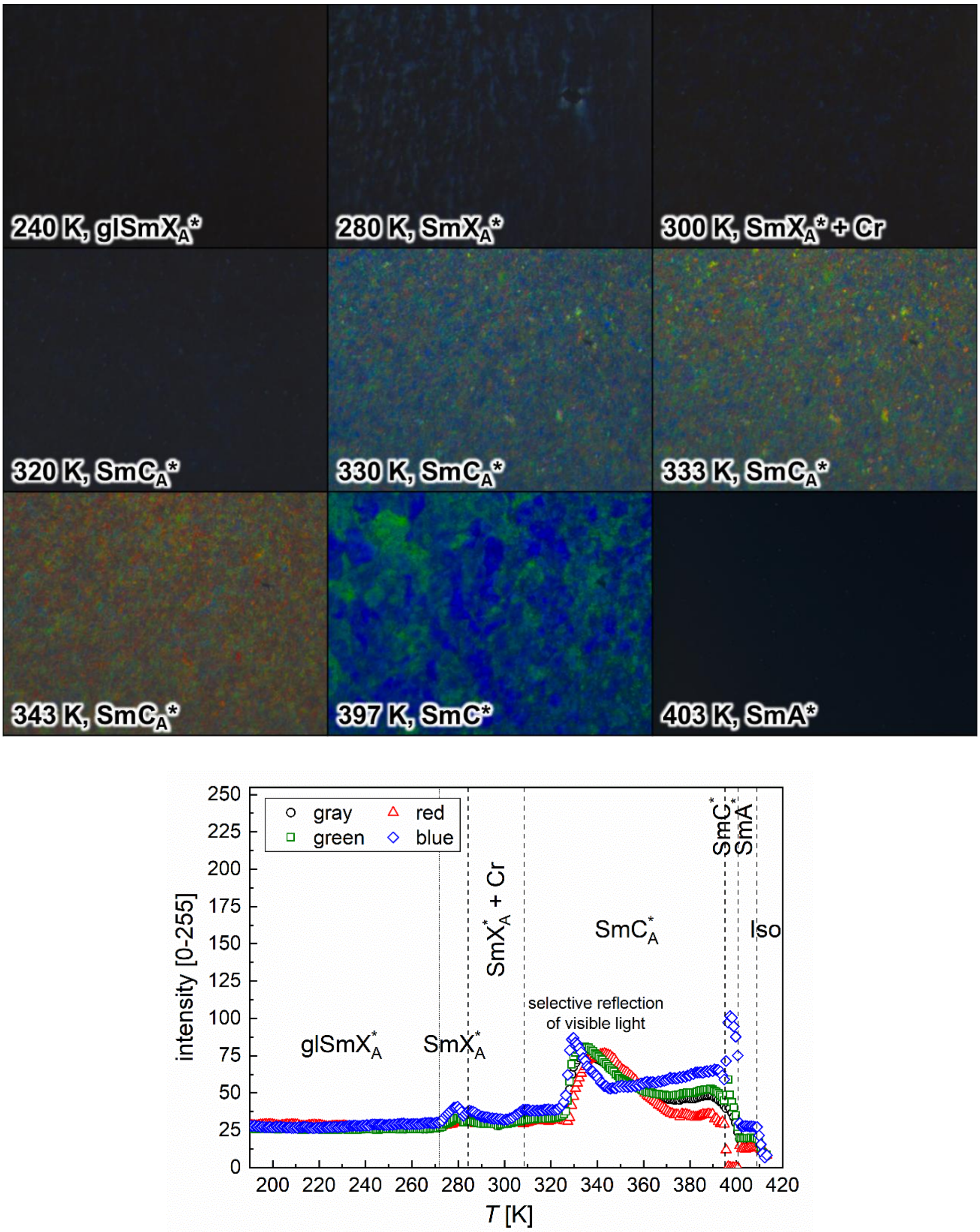

Figure S17. POM textures (622 × 466 μm^2) of MIX23HH6 collected at the 10 K/min heating rate in the reflection mode. The plot below shows the red, green, blue components and weighted total intensity of each texture.

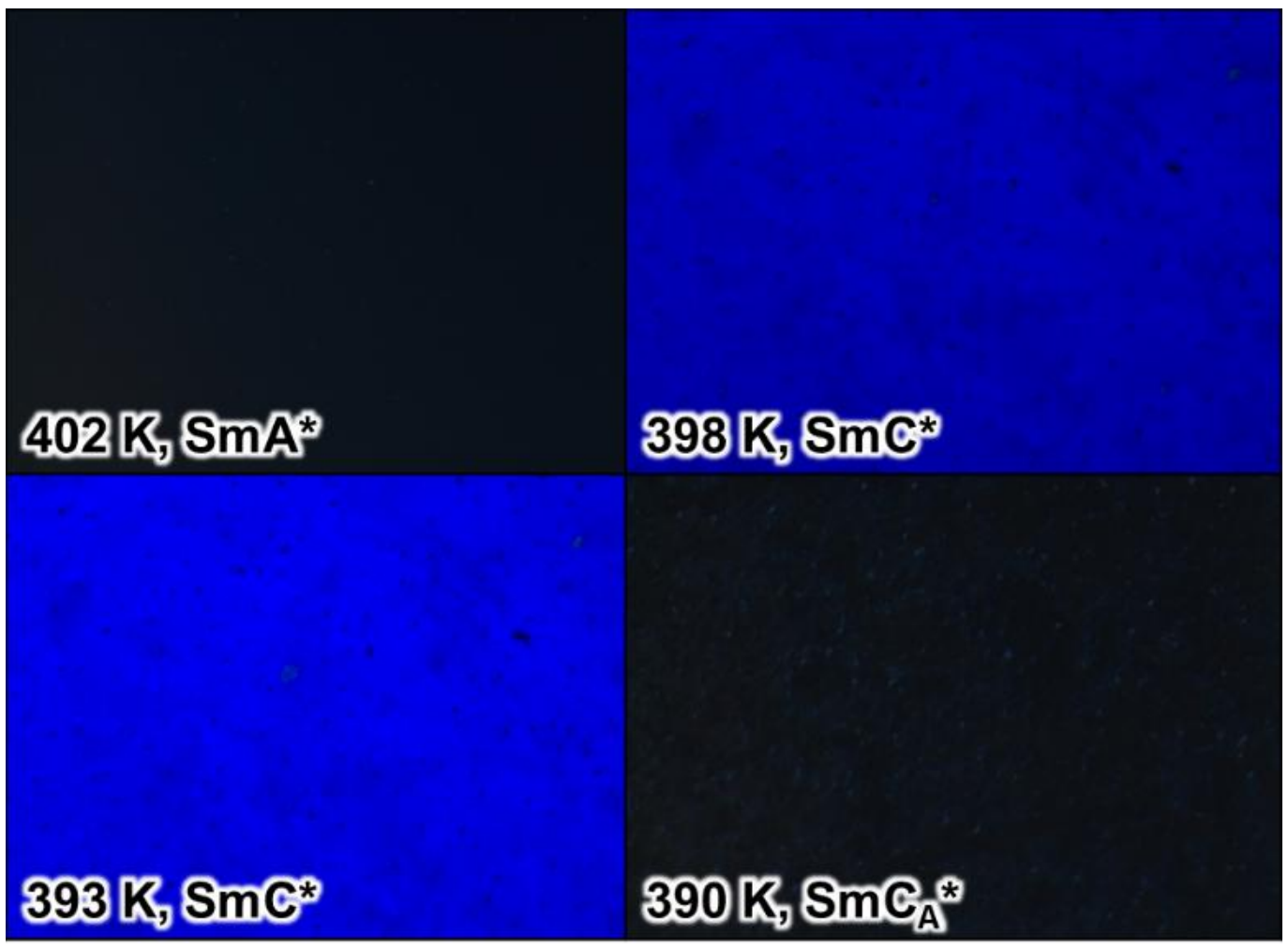

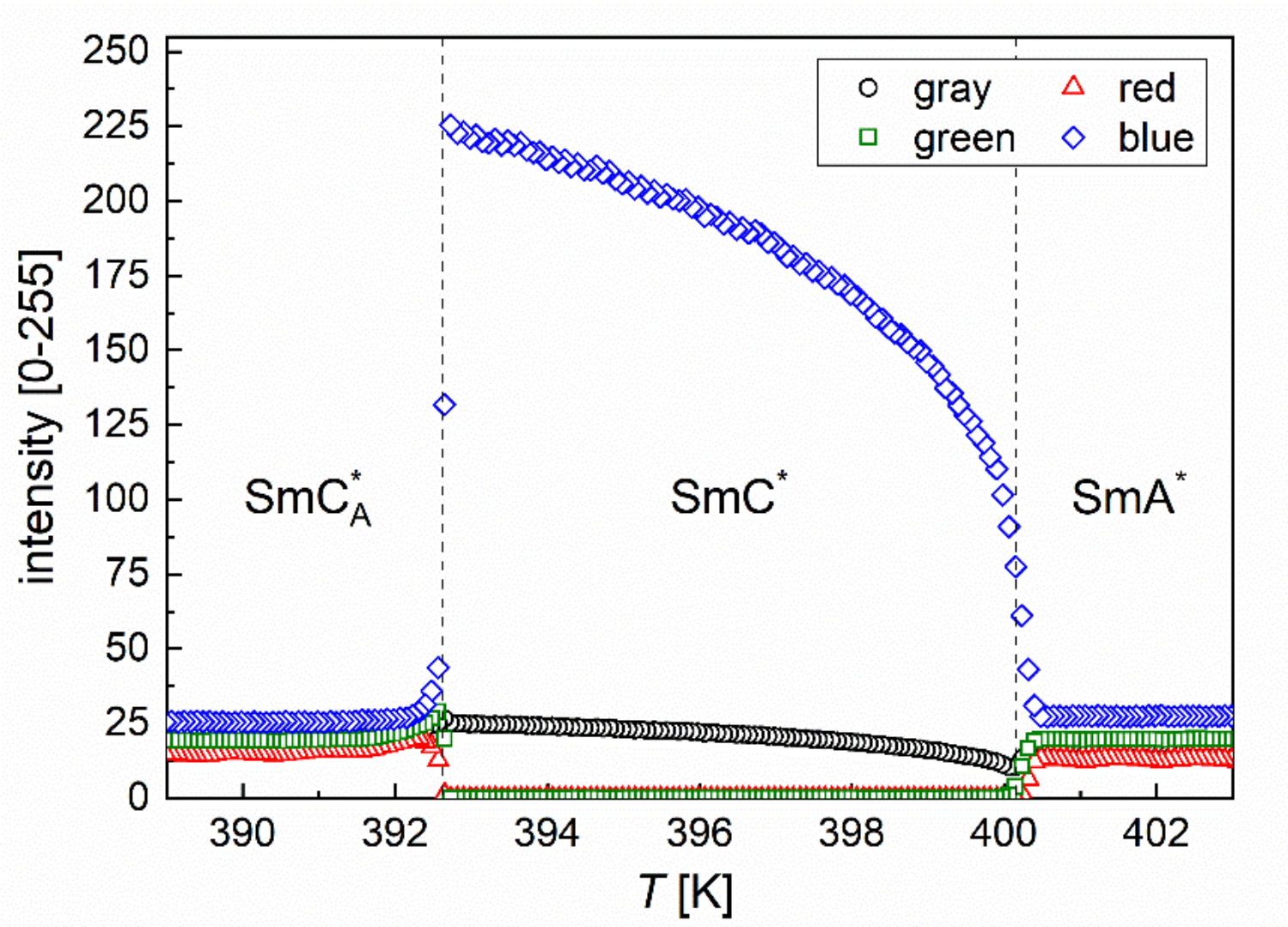

Figure S18. POM textures (622 × 466 μm^2) of MIX23HH6 collected at the 1 K/min cooling rate in the reflection mode. The plot below shows the red, green, blue components and weighted total intensity of each texture.

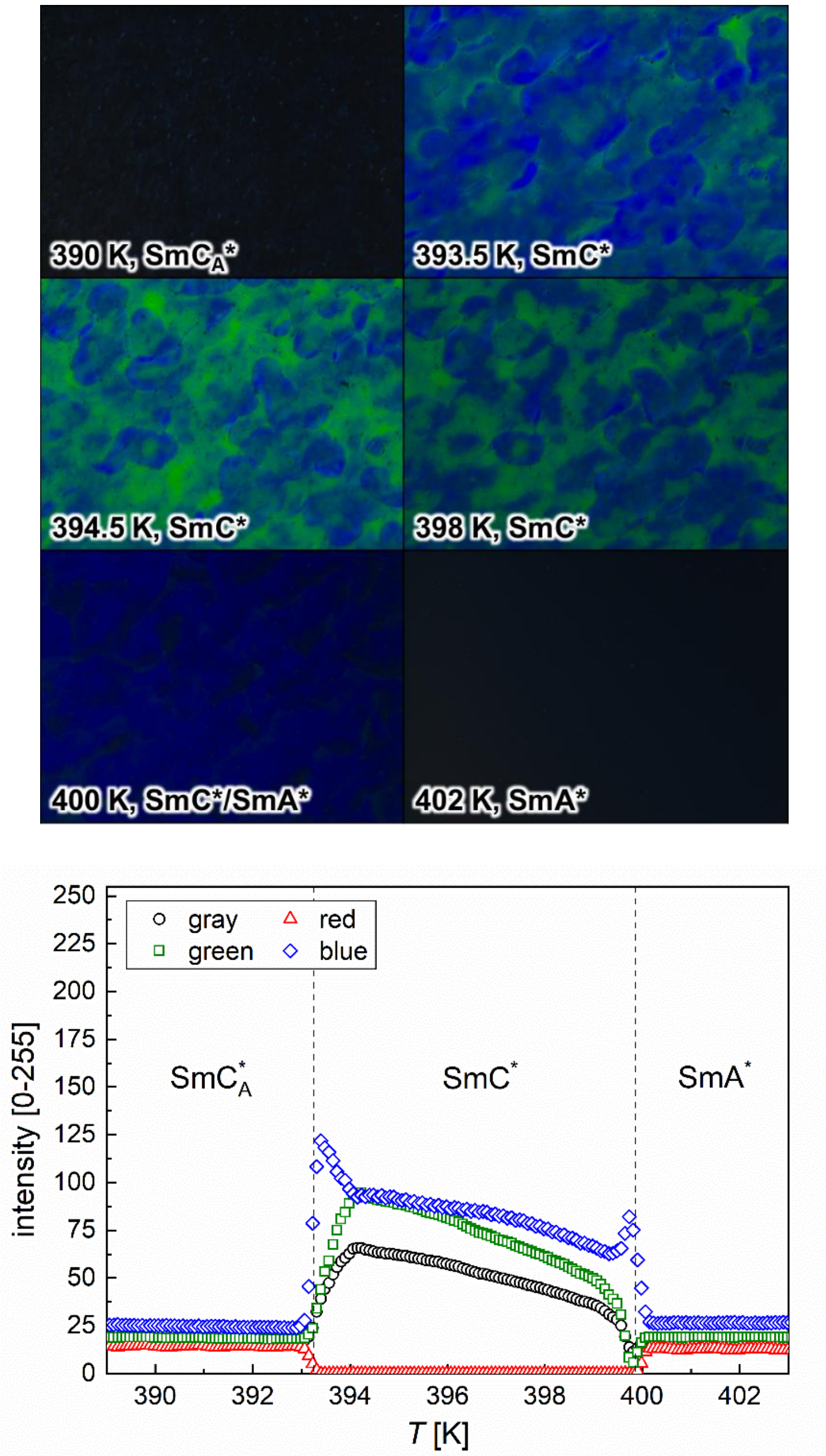

Figure S19. POM textures (622 × 466 μm^2) of MIX23HH6 collected at the 1 K/min heating rate in the reflection mode. The plot below shows the red, green, blue components and weighted total intensity of each texture.
